# Bayesian graph convolutional network with partial observations

**DOI:** 10.1371/journal.pone.0307146

**Published:** 2024-07-18

**Authors:** Shuhui Luo, Peilan Liu, Xulun Ye

**Affiliations:** 1 Faculty of Business, University of Nottingham Ningbo China, Ningbo, Zhejiang, China; 2 College of Science and Technology, Ningbo University, Ningbo, China; 3 Institute of Computer Science and Technology, Ningbo University, Ningbo, China; Stockholms Universitet, SWEDEN

## Abstract

As a widely studied model in the machine learning and data processing society, graph convolutional network reveals its advantage in non-grid data processing. However, existing graph convolutional networks generally assume that the node features can be fully observed. This may violate the fact that many real applications come with only the pairwise relationships and the corresponding node features are unavailable. In this paper, a novel graph convolutional network model based on Bayesian framework is proposed to handle the graph node classification task without relying on node features. First, we equip the graph node with the pseudo-features generated from the stochastic process. Then, a hidden space structure preservation term is proposed and embedded into the generation process to maintain the independent and identically distributed property between the training and testing dataset. Although the model inference is challenging, we derive an efficient training and predication algorithm using variational inference. Experiments on different datasets demonstrate the proposed graph convolutional networks can significantly outperform traditional methods, achieving an average performance improvement of 9%.

## 1 Introduction

Recent years have witnessed the great success of Convolutional Neural Network (CNN) in many different data processing fields [1–4]. However, CNNs are primarily designed for the grid dataset. Graph, as one of the most widely used non-grid data structure in the modern digit society (such as community detection, drug design, molecular generation, and etc. [5–9]), reveals its difficulty when exploiting the CNN architecture. To overcome this difficulty, Graph Convolutional Networks (GCNs) [10–12] have been proposed. In a typical GCN framework, graph is organized into two different parts, node relationship *A* and node feature *X*. Then, a deep network framework is applied to map the node features *X* into a novel graph representation space constrained by the graph relationship *A* [13, 14].

**Fig 1:**
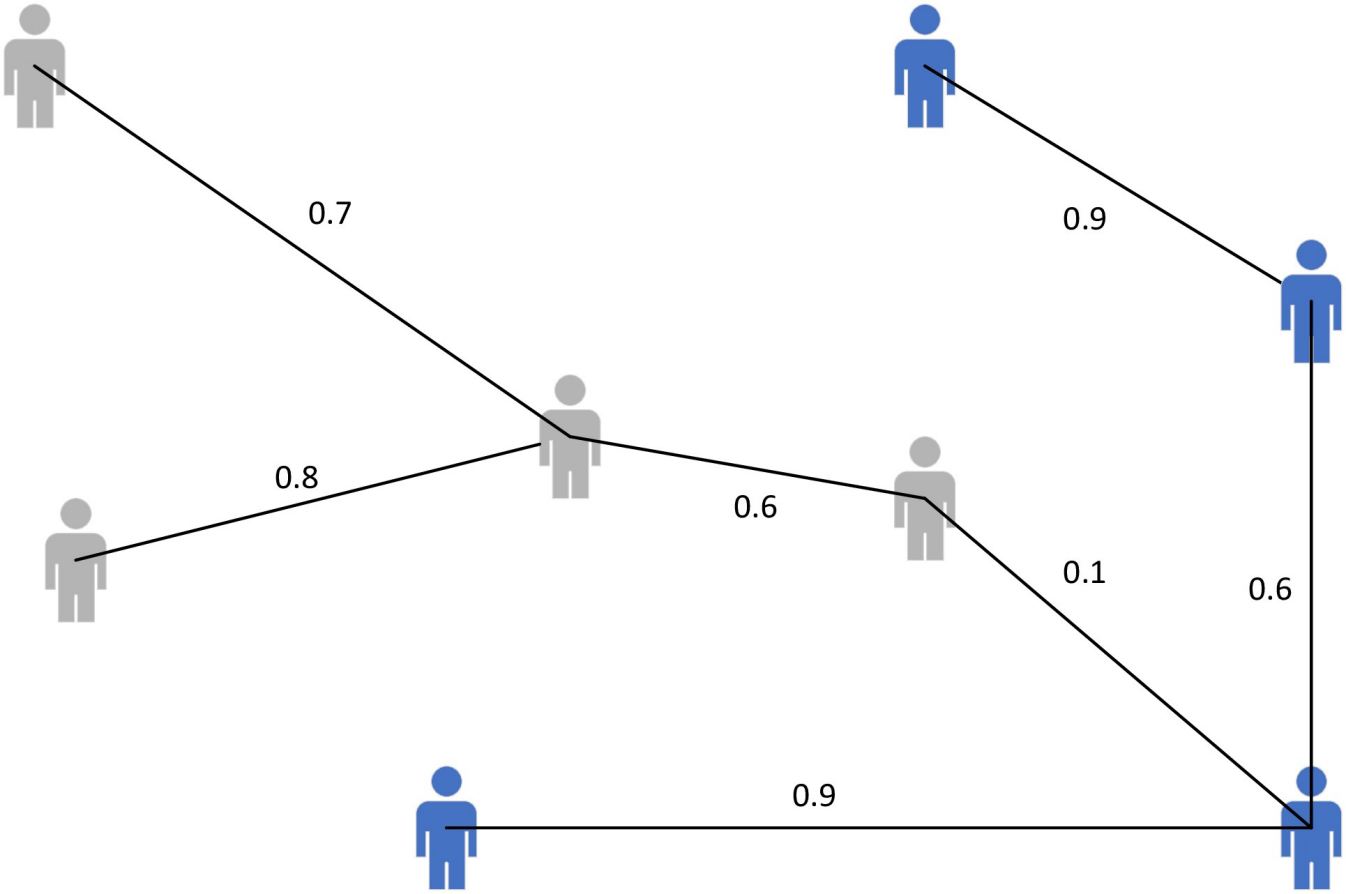
Private social network. In this figure, there are two communities (each color indicates one community). In this network, due to the privacy considerations, nodes have no information to display.

Although the existing GCNs are powerful and effective tools, it assumes that the graph nodes are equipped with fully observed features *X*. This assumption may not hold in many real applications (Fig 1, in a private social network, node has no information to display.), which have only the relationships and the corresponding node features are unavailable. To tackle this problem, first, we construct a Bayesian GCN generative model, in which the pseudo features are used to simulate the real features (when applying our method to the graph with features, a concatenation strategy is proposed and constructed in the pseudo features generation process). Then, a hidden structure term is proposed to generate suitable pseudo features. Finally, we derive the corresponding training and predication algorithm. We conclude our main contributions as follows:

- Conventional GCN has been extended to a Bayesian framework, where pseudo features generated from the stochastic process are used to simulate real features. Our model handles GCN graph processing task with or without node features in a unified framework, offering a novel prospect within a Bayesian framework for the graph node classification without features.
- To maintain the independent and identically distributed property of the pseudo features, a hidden space structure preservation term has been proposed and utilized to constrain the sample generation process.
- For the non-conjugated property, we employ a mean field variational inference integrated with Variational Auto-Encoder (VAE) for model training and predication.

We organize our paper as follows. Section two covers the related work. Section three will briefly reviews the preliminary knowledge. Section four presents the details of the proposed method and the corresponding variational inference algorithm. Section five is the experimental results, including some parameter effect analysises are also carried out in this section. Section six concludes the paper.

## 2 Related work

### 2.1 Node classification

Generally, GCN can be roughly organized as two categories: spatial methods and spectral methods. In the first category, graph convolution is defined as the operation of neighbors [15, 16]. For example, Atwood et al. [15] extend the Convolutional Neural Networks by employing the graph diffusion process to integrate the node neighbor information. Duvenaud et al. [16] introduce the graph convolutional operation by applying the convolution-like propagation rule on graphs. Niepert et al. [17] define the GCN by converting the graph into sequences and apply the conventional CNN model. Monti et al. [18] present mixture model CNNs, then they define a CNN model on the graph data. In the second category, spectral representation of graphs is introduced into the graph convolution definition [19]. For example, Bruna et al. [19] construct the graph convolutional operation in the Fourier domain by exploiting the eigen-decomposition of graph Laplacian matrix. Due to the high computational complexity, Defferrard and Kipf et al. [20, 21] extend Bruna’s work by approximating the spectral filters with the Chebyshev expansion and the first-order approximation of spectral graph. Jiang, Tang, Li and Franceschi et al. [13, 14, 22–24] improve the GCN classification accuracy by utilizing a graph learning framework. Gan and Zhao et al. [25, 26] generate multiple graph structures and fuse the information of multiple graphs to improve the GCN performance. Besides these theoretic analysis, many researchers focus on extending the GCN to the conventional machine learning and computer vision tasks. For example, Cai et al. [27] exploit GCN to estimate the 3D Pose. Yan and Huang et al. employ GCNs to handle the skeleton-based action recognition [28–30]. Yang and Wang et al. [31–34] extend GCN application scope to the clustering task. Zhang and Chen et. al. exploit graph convolutional network for the zero-shot learning [35–37]. Compared to these conventional GCN models which require the node feature, our model could handle the graph data without this information.

### 2.2 Incomplete data learning

Data missing is a ubiquitous issue which has attracted many attentions in the field of data mining, machine learning and computer vision [38]. When handling this problem, the most widely studied method is the data imputation [39], which fills the missing attribute values with the help of the known attribute. For example, Azur et. al. [40] use chained equations to iteratively impute the miss variables in feature. Mean methods impute the miss values by averaging the known values [38], or discard the corresponding missing values directly to make the algorithm work [41]. Although these methods have shown its effective, the assumptions they make when constructing this method might result in biased predictions [42]. Besides these methods which take the statistical views, recently, many researches refer to the machine learning technics. For example, Acuna et. al. [43] exploit the k-nearest neighbors algorithm. Dick et. al. [44] apply the generative model to infer the missing values. Lakshminarayan et. al. [45] use the decision trees. Zhang et. al. exploit associate-rule based imputation and rough set method [46, 47]. More recently, deep neural networks have also been applied to the data missing imputation problems [48–51]. Although these methods have achieved the remarkable performance in many data imputation tasks. They take the assumption that the features can be partially observed. Unlike these methods, our method can be applied to the graph node classification problem without features.

### 2.3 Deep generative models

Deep generative models aim to model the real complicated distributions via a deep neural network. Generally, deep generative model can be roughly categorized into two different classes. The first broad class is the variational inference and sampling based method. For example, Variational Auto-Encoders (VAEs) [52, 53] extend the Gaussian generative model with the deep neural network [54–56], and then use the variational inference to compute the posterior probability and the likelihood function. Deep Belief Networks (DBNs) [57, 58] stack multi-layer restricted Boltzmann machines [59] and apply the sampling based method to achieve the maximized likelihood probability. In addition to the first class method which learns the model with the variational inference and sampling method, the second type of deep generative model is the implicit method. The most typical method is the Generative Adversarial Network [60, 61], which expands the maximum likelihood principle of the generative model by using an adversarial strategy. Stochastic network is another implicit method which uses Markov chain to construct the deep generative model [62]. Different from the above methods which use the generative model to fit the real distributions of the given samples, our model is designed to graph node classification task with partial observed graph data.

## 3 Preliminary

In this section, we briefly review the preliminary knowledge of Graph Convolutional Network (GCN) and Variational Auto-Encoder (AVE) model. Our model will be derived in a later section.

### 3.1 Graph Convolutional Network

Graph Convolutional Network (GCN) extends the conventional convolutional operation on the grid structure to non-grid structure within the deep neural network framework. Given a graph denoted as (*A*, *F*) where *A* encodes the pairwise relationship and *F* represents the node features, GCN employs the following layer-wise propagation in hidden layers:

$$H_{h}=\sigma \left(D^{-1/2}AD^{-1/2}H_{h-1}W_{h-1}\right)$$

where *H*_*h*−1_ and *H*_*h*_ are the hidden output in the layer *h* − 1 and *h* (*H*_0_ is set to be *X*). *D* is a diagonal matrix with $d_{i}=\sum_{j=1}^{N}A_{i,j}$. *σ*(⋅) is the activation function which is usually set as the ReLU or the sigmoid. For classification task, GCN defines a softmax convolution layer at the final output layer.

$$Y=softmax(D^{-1/2}AD^{1/2}t_{H}W_{H})$$

where *Y* is usually used as the label distribution. For the training process, GCN utilizes the cross-entropy loss.

### 3.2 Variational Auto-Encoder

Variational Auto-Encoders (VAEs), introduced by Kingma and Welling [52], are popular methods in many machine learning and computer vision tasks. Given the observation dataset $\hat{X}={\left\{{\hat{x}}_{n}\right\}}_{n=1}^{N}$, VAE assumes the model can generate the data as follows: (1) For the hidden variable *t*_*i*_, draw *t*_*i*_ ∼ *N*(*t*|0, *I*).

(2) Draw the observation samples ${\hat{x}}_{i}∼N\left(x|F\left(t,W\right),\sigma \right)$. where *t*_*i*_ is the hidden variable, *F*(*t*, *W*) is a neural network. To train the network, VAE uses the variational inference and derive the following loss function:

$$\begin{array}{c} \begin{array}{c} \int q\left(t_{i}\right)\;\text{log}\;\{N\left({\hat{x}}_{i}|F\left(h_{i}|O\right)\right)\frac{N(h_{i}|0,I)}{q(t_{i})}\}dt_{i} \end{array} \end{array}$$ (1)

where *q*(*t*_*i*_) is also a neural network. Then, VAEs optimize the above loss function via a standard back-propagation algorithm integrated with a reparameterization trick. Following, we will use the same optimization trick, but use different notations of ${\hat{x}}_{i}$ and *q*(*t*_*i*_).

## 4 Our proposed method

In this section, we introdude our method called Bayesian Graph Convolutional Network (BGCN) for graph node classification without features. Subsequently, we derive the corresponding training and predication algorithm based on variational inference. Main notations and descritions are summarized in Table 1.

**Table 1:** MAIN notations and descriptions.

NOTATIONS	DESCRIPTIONS
*A*	Graph pairwise relationship
*F*	Node features
*Y*	Training lables
*N*	Node number
*D*	Feature dimension of a given node
*M*	Number of training samples
*x*_*i*_	Pseudo feature
*N*(⋅)	Gaussian distribution
*GCN*(*x*, *W*)	Graph convolutional network with feature *x* and weight *W*
*p*(*Y*, *A*|0, *I*, *w*)	Joint distribution of observation Y and A
*q*(⋅)	Variational posterior distribution
Σ(*x*_*i*_), *u*(*x*_*i*_)	Output of $q_{\tilde{w}}\left(x_{i}\right)$
*Tr*()	Trace of a matrix
*S*	Sampling times of the $x_{i,s}^{*}$
*Au*_*i*_, *As*_*i*_	Auxiliary variable *Au*_*i*_ = *u*(*x*_*i*_), *As*_*i*_ = Σ(*x*_*i*_)
*L*	Laplacian matrix of *A*

### 4.1 Bayesian graph convolutional network

**Fig 2:**
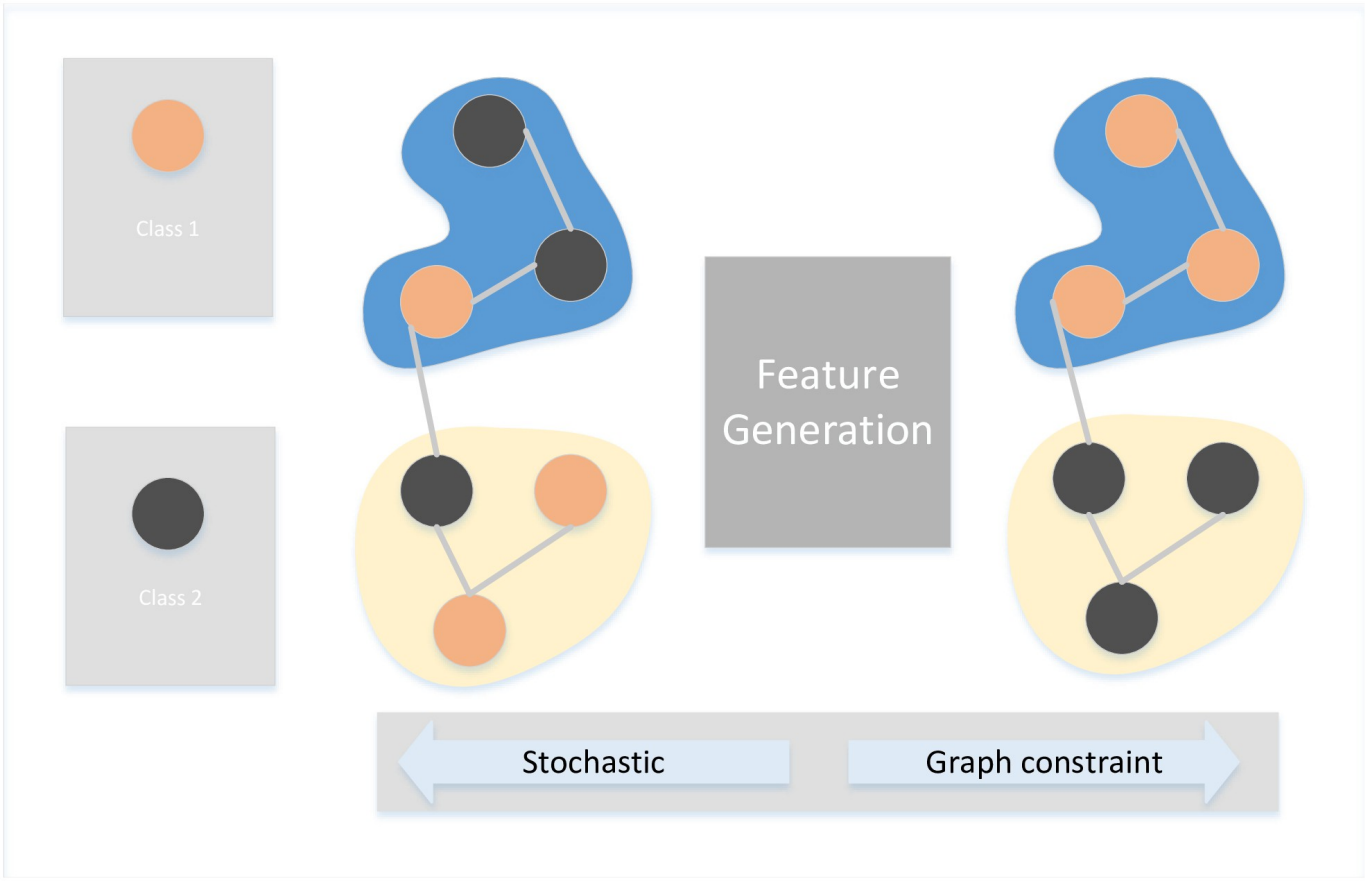
Feature generation problem. The figure illustrates the pseudo features generated by two different distributions: random distribution and graph-constrained distribution. Pseudo features generated by the random distribution fail to preserve class relations (same class in different space.). However, pseudo features generated with graph constraints successfully maintain the class relationships during the generation process (same class in the similar space.).

**Fig 3:**
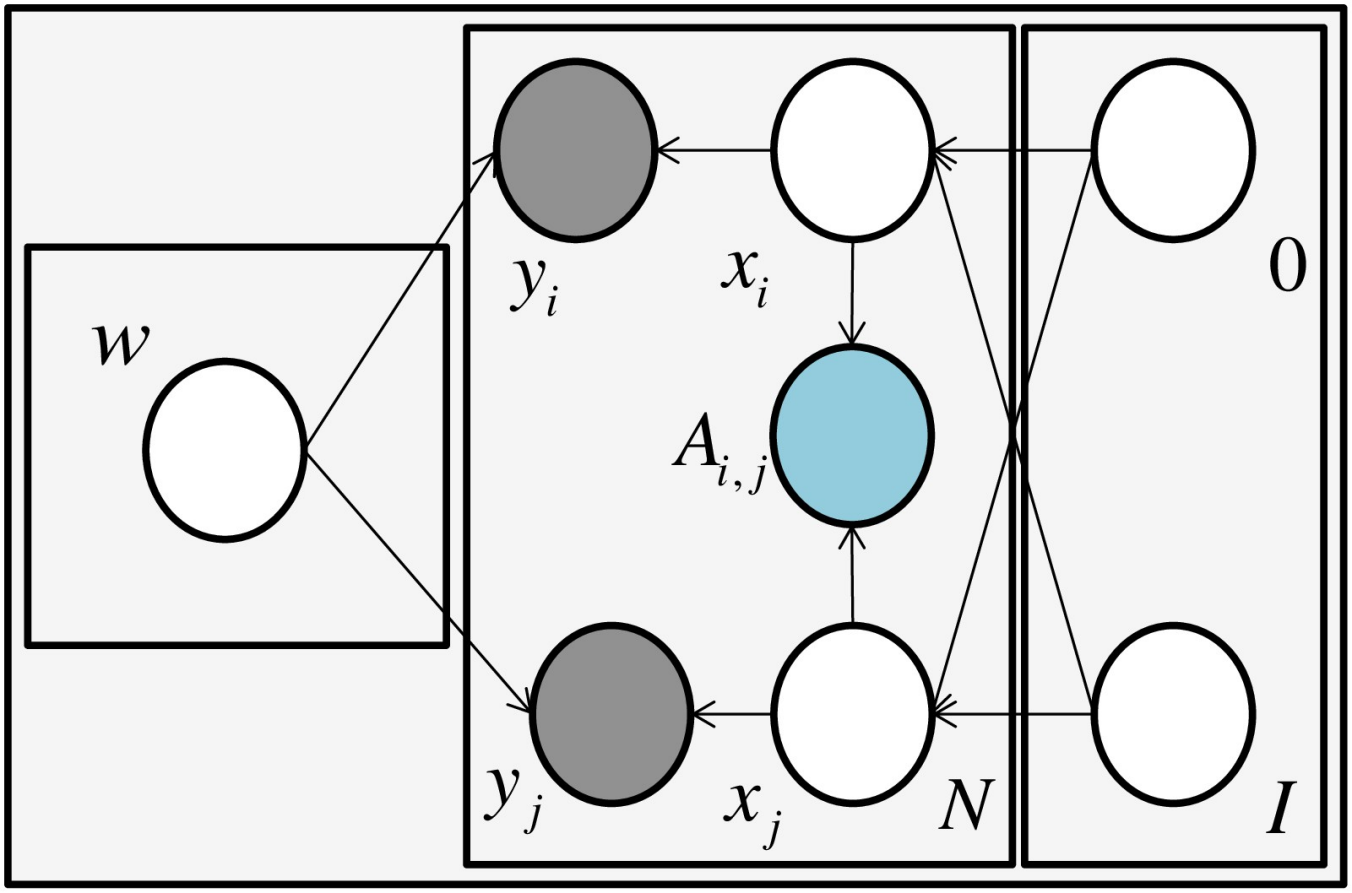
Dependency between random parameters in our model. Probabilistic graphical model of BGCN. Specifically, consider the graph associated with the model, denoted as G. In this graph, blue nodes represent observations, while gray nodes correspond to partial observation labels. Notably, from the figure, we observe that our observed label *Y* is generated from the pseudo feature *x*_*i*_.

Given the graph (*A*, *F*, *Y*) where $A\in R^{N\times N}$, $F\in R^{N\times D}$ and $Y\in R^{M\times K}$ denote the pairwise relationship, node features the and the training labels respectively. Here, *M* denotes that there is *M* training samples in the graph (*A*, *F*)(*M* < *N*), *K* is the class number. we consider the problem that *F* is not available. In order to handle this problem, our idea is to equip the input with pseudo features. One straightforward pseudo feature is a constant value. However, this leads a problem that the input is unable to distinguish the difference between different samples. Another idea is that the pseudo features are generated from random distributions. Although random pseudo features can identify the difference, features from training set and testing set come from different distribution (this refers to the problem of nonindependent and nonidentically distributed issue, Fig 2). To tackle this problem in our model, we use the graph to constrain the pseudo feature generation process, which requires the pseudo features are generated with the consistent of the given graph. Note that pseudo features can be used to handle node without features, when the node features are available, we concatenate it with the generated pseudo feature. Our BGCN generation process is: (1) For the pseudo feature *x*_*i*_, *x*_*j*_, draw *x*_*i*_, *x*_*j*_ ∼ *N*(*x*|0, *I*). (2) Maintain the struture of *x*_*i*_, *x*_*j*_ with the graph relationship *A*, draw *l*_*i*,*j*_ ∼ *N*(*l*|*A*_*i*,*j*_|*x*_*i*_ − *x*_*j*_|^2^, *σ*). (3) For labels of the pseudo features *y*_*i*_, *y*_*j*_, draw *y*_*i*_, *y*_*j*_ ∼ *N*(*y*|*GCN*(*x*, *W*), *σ*). where *N*(*x*|0, *I*) is the Gaussian distribution with the constant parameter 0 and *I*. *A*_*i*,*j*_ is the element in *A*. *GCN*(*x*, *W*) denotes the Graph Convolutional Network with the parameter *W*. Note that, when maintaining the structure in the hidden space, we set *l*_*i*,*j*_ = 0, which means that the generated pseudo features *x*_*i*_ and *x*_*j*_ are forced to be consistent with the graph structure. The Probabilistic Graphical Model (PGM) is shown in Fig 3 where the pseudo features are generated from the Gaussian distribution and constrained by the graph, and the labels are generated from the pseudo features. Our model alters the discriminative GCN model to a generative model.

### 4.2 Variational inference

In the previous section, we have constructed the corresponding Bayesian graph convolutional network model. In this section, we derive the corresponding learning and predication algorithm. Following the variational inference framework [63], we derive the Evidence Lower BOund (ELBO):

$$\begin{array}{c} \begin{array}{cc} & \text{log}\;p(Y,A,|0,I,w)\geq \int q\left(X\right)\;\text{log}\;\frac{p(X,Y,A)}{q(X)}dX\\ & =\sum_{i,j=1}^{N}\int \{q\left(x_{i}\right)\;\text{log}\;N\left(y_{i}|GCN\left(x_{i},W\right),\sigma \right)+\\ & q\left(x_{i},x_{j}\right)\text{log}\;N(l_{ij}|A_{i,j}|x_{i}-x_{j}{|}^{2},\sigma )+q\left(x_{i}\right)N\left(x_{i}|0,I\right)\} \end{array} \end{array}$$ (2)

where *p*(*Y*, *A*, |0, *I*, *w*) denotes the joint distribution of observations *Y* and *A*, *p*(*X*, *Y*, *A*) is the joint distribution of the hidden variables. *q*(*X*) represents the variational posterior distribution of the pseudo feature distribution. Note that, since label *Y* is generated from the pseudo feature with the GCN network, *q*(*X*) cannot be derived following the standard variational framework. In our model, we adopt a strategy used in the Variational Auto-Encoder (VAE) [55] network, in which the hidden variable *X* can be form as another neural network with parameter $\tilde{w}$, $q\left(X\right)=\prod_{i=1}^{N}q_{\tilde{w}}\left(x_{i}\right)$.

We now extend the ELBO, and derive the following loss function:

$$\begin{array}{c} \begin{array}{cc} & \text{max}\{-Tr\left(\Sigma \left(x_{i}\right)\right)+E_{q}(\text{log}\;N\left(Y|GCN\left(X,w\right),\sigma \right))\\ & +\text{log}|\Sigma \left(x_{i}\right)\left|-1/\sigma A_{i,j}(Tr(\Sigma \left(x_{i}\right)\right)\\ & +Tr\left(\Sigma \left(x_{j}\right)\right)+|u\left(x_{i}\right){|}^{2}+{\left|u\left(x_{j}\right)\right|}^{2}-2u{\left(x_{i}\right)}^{T}u\left(x_{j}\right))\} \end{array} \end{array}$$ (3)

where Σ(*x*_*i*_) and *u*(*x*_*i*_) are the output of the $q_{\tilde{w}}\left(x_{i}\right)$, in which the first half of $q_{\tilde{w}}\left(x_{i}\right)$ forms as the mean and the last half forms as the covariance. Note that, integrating over the neural network has no analytical solution. Thus, we employ a sampling method to calculate this term.

$$\begin{array}{c} \begin{array}{cc} & \int q\left(x_{i}\right)\{\text{log}\;N(y_{i}|GCN\left(w,x_{i}\right),\sigma )\}\\ & =\frac{1}{S}\sum_{s=1}^{S}\text{log}\;N(y_{i}|GCN\left(w,x_{i,s}^{*}\right),\sigma ) \end{array} \end{array}$$ (4)

where $x_{i,s}^{*}$ is sampled from the distribution $q_{\tilde{w}}\left(x_{i}\right)$; for the parameter *σ*, we set it as 2. Optimizing Eq 4 can be done using the standard back-propagation algorithm if the feature is available. However, this violates our assumption and also leads to a trivial solution. A simpler method is that we take the derivative with respect to the parameter Σ(*x*_*n*_) and *u*(*x*_*n*_) directly. But taking derivative w.r.t. a neural network is challenging, and optimizing through a neural network is inefficient. Below, we show, by using some simple constraints and auxiliary variables *Au*_*i*_ = *u*(*x*_*i*_), *As*_*i*_ = Σ(*x*_*i*_), our method can achieve the efficient solution. From the ELBO, we know that loss function to optimize *u*(*x*_*i*_)_*i*_ is:

$$\begin{array}{c} \begin{array}{cc} & \text{min}\;1/\sigma A_{i,j}\{Au_{i}^{2}+Au_{j}^{2}-2Au_{i}^{T}Au_{j})\} \end{array} \end{array}$$ (5)

We set some constraints to the variable *u*(*t*_*i*_), that is *AuAu*^*T*^ = *I*:

$$\begin{array}{c} Au=\{Au_{1},\ldots ,Au_{i},\ldots ,Au_{N}\} \end{array}$$

Rearranging the above equations, we have:

$$\begin{array}{c} \begin{array}{cc} & \text{min}\;1/\sigma Tr\left(AuLAu^{T}\right),s.t\;AuAu^{T}=I,\;Au=U \end{array} \end{array}$$ (6)

Where *U* is defined same as *Au*. To solve this problem, we form the Lagrangian function, set $\hat{\sigma }=2$ and set λ_*d*_ as the Lagrangian multipliers of the first constraint and relax *AuAu*^*T*^ = *UU*^*T*^, Then by taking the derivative w.r.t. *Au*_*d*_:

$$\begin{array}{cc} & LAu_{d}^{T}-\left(\lambda_{d}+\lambda_{u}\right)Au_{d}^{T}=0 \end{array}$$

Where *L* is the graph Laplacian of *A*. *Au*_*d*_ is *d*’th row of *Au*. λ_*u*_ is the Lagrangian multipliers of the second constraint. The equation above can be solved by employing eigenvalue decomposition. For *Us*_*n*_, we have:

$$\begin{array}{c} \begin{array}{cc} & Us_{n}^{-1}=\sum_{k=1}^{K}I+\sum_{j=1}^{N}1/\sigma A_{i,j}I \end{array} \end{array}$$ (7)

**Fig 4:**
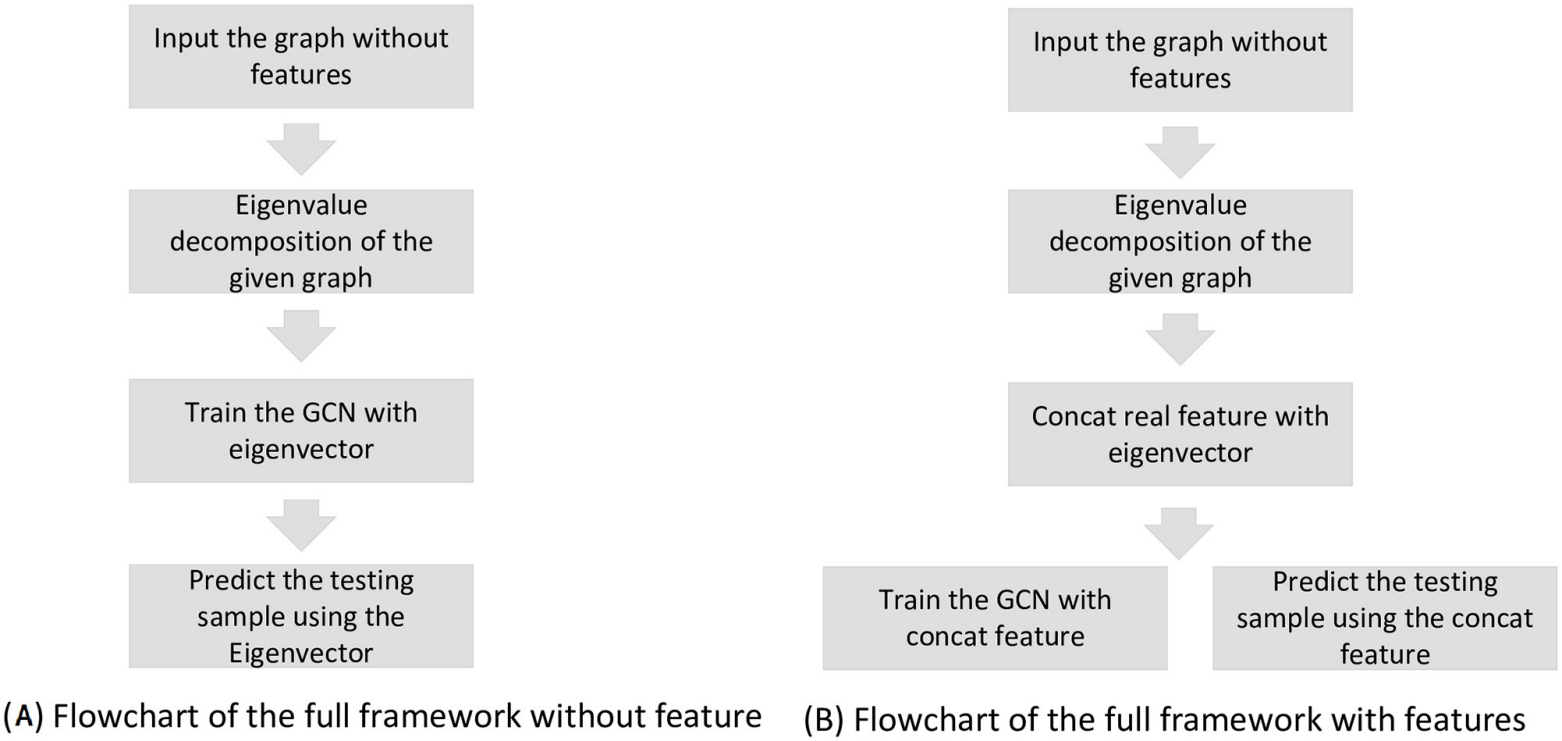
Flowchat of the proposed algorithm. Figure (A) demonstrates the flowchart of the proposed method without node features. Figure (B) is the flowchart of the proposed method with node features.

**Fig 5:**
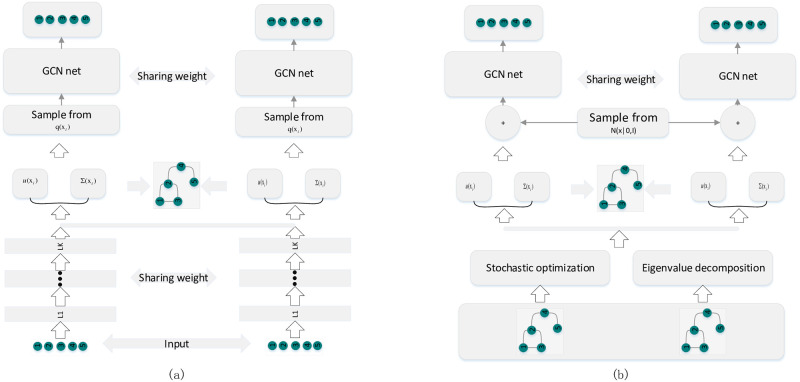
BGCN optimization framework. In figure (a), we take the node feature as the input and use a neural network to infer the posterior distribution. However, since we sample $x_{i,m}^{*}$ from a neural network variational posterior distribution $q_{\tilde{w}}\left(x_{i}\right)$ with input *F*, the entire algorithm cannot be optimized. In figure (b), instead of optimizing the network with feature *F*, we apply eigenvalue decomposition and an updating rule to achieve the mean and covariance of the output using only the graph *A*. When applying the full features *F*, we concatenate the variational posterior parameter with the given features.

After achieving the initialized *u*(*x*_*i*_)_*i*_, we exploit the standard back propagation algorithm to further optimize $\text{log}\;N(y_{i}|GCN\left(w,x_{i,m}^{*}\right),\hat{\sigma })$. We summarize the BGCN training and predicating algorithm in algorithm 1. Flowchart of the proposed method is summarized in Fig 4. The Full optimization procedure is summarized in Fig 5.

**Algorithm 1** Training and predication algorithm for BGCN with fully observed features

**Require**:

 Labels $Y\in R^{M}$ for the training dataset, a given graph *A*, and the corresponding features *F*.

**Ensure**:

 Labels $Y\in R^{M}$ for the predication dataset.

 **Training procedure**

 1: Compute the parameters of *q*(*X*) using Eqs (6) and (7).

 2: Sampling the parameters $x_{i,m}^{*}$ from $q_{\tilde{w}}\left(x_{i}\right)$ with the training dataset.

 3: Normalizing the parameter *u*(*x*_*i*_).

 4: If the the observed feature *F* is available, concating normalized parameter *u*(*x*_*i*_) with the observed feature *F*. Else, use parameter *u*(*x*_*i*_) as the feature.

 5: Using the loss of Eq 4 to train the GCN model.

 **Predication procedure**

 1: Sampling $x_{i,m}^{*}$ from $q_{\tilde{w}}\left(x_{i}\right)$ with the given predication dataset.

 2: Normalizing the parameter *u*(*x*_*i*_).

 3: If the the observed feature *F* is available, concating normalized parameter *u*(*x*_*i*_) with the observed feature *F*. Else, use parameter *u*(*x*_*i*_) as the feature.

 4: Using the Eq (4) to achieve the GCN output.

Note that, when our model is applied to the graph dataset with the node features, from the derivation, we know that concating the original features with the pseudo features is equal to concat it with the posterior parameter *u*(*t*_*i*_). For the computational cost, The main computational cost comes in two branches: (1) eigenvalue decomposition, which adds the *O*(*N*^3^) where *N* stands for the number of graph nodes; (2)GCN model. Suppose that, in the GCN model (with *L* layers and *K* iterations), each node has *m*_*l*_-dimensional features. Then, computational cost is $K\times \sum_{l=1}^{L}O\left(N^{2}m_{l}\right)+O\left(N^{3}\right)$.

## 5 Experiments

In this section, we empirically evaluate the effectiveness of the proposed BGCN, and compare it to several existing methods. Then, we measure the influence of BGCN parameters on real graph datasets.

### 5.1 Experimental setup

#### Datasets

Three real-world graph datasets are used to evaluate our method performance, including the Citeseer, Cora and Pubmed [64]. The details of these datasets are as follows:

(1) Citeseer Dataset: Citeseer is a citation network which contains 3327 nodes, 4732 edges and six classes.
(2) Cora Dataset: Cora dataset has 2708 nodes and 5429 edges, in which every node falls into 6 classes.
(3) Pubmed Dataset: Pubmed is a dataset with 19717 nodes and 44338 edges. Each node in the dataset falls into 3 classes.

**Fig 6:**
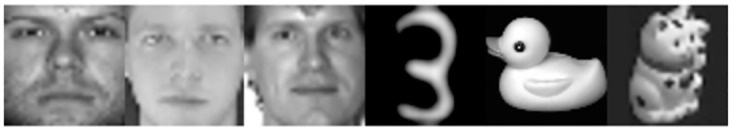
Illustration of some samples from six image datasets.

In addition to the real graph datasets, we also exploit several image datasets (Extended YaleB, Orl, Yale, Usps, Coil20, Coil100), in which we use the *k*-nearest neighbor method to construct the graph. Details about neighbors and attribute used in these image datasets are demonstrated in Table 2. Some samples from the six image datasets are demonstrated in Fig 6.

**Table 2:** Attribute of the image dataset along with the neighbors used.

Datasets	Neighbors	Classes	Images
Extended YaleB	5	38	2432
Orl	5	40	400
Yale	5	15	11
Usps	15	10	9298
Coil20	15	20	1440
Coil100	5	100	7200

#### Experimental settings

For citeseer, cora and pubmed, we follow the experimental settings in [21]. For the Coil20 dataset, we use 30 samples each class as the training dataset, and use the other 42 samples as the testing dataset. For the usps dataset, we use 200 samples each class as the training dataset, and the other samples as the testing dataset. In the case of the Extended YaleB, Yale, and Orl datasets, we split the samples evenly, using half for training and the other half for testing. For all datasets, we maintain a learning rate of 0.01 and a dropout rate of 0.5. In our experiment, we also use the *l*_2_ regularization item for the weight decay. The loss function in our experiment is altered to cross entropy which is equal to the least square loss function used in our model. We set the hidden layer in our experiment with 16-dimension features, and 3 layers. For the he evaluation metrics, we exploit the classification accuracy (the proportion of correctly predicted instances out of the total number of instances in the dataset). We implement our model on a computer with XEON 4210R CPU and 62GB RAM. The GPU is RTX 2080TI with 11GB memory. GCN is implemented with tensorFlow. The system we used in our experiment is Linux (Ubuntu version).

#### Baselines

In our experiment, we compare our method with some other graph based learning algorithms. The compared methods contain: 1) Label Propagation (LP) [65], 2) DeepWalk network [66], 3) The original Graph Convolutional Network [21], 4) Chebyshev polynomial version of graph convolutional network [20], 5) Graph attention networks (GAT) [67]. Note that, for the graph based deep learning methods like GCN, GAT and Chebyshev, we replace the input with noise input (input generated from a Gaussian distribution) and non-information input (input with some constant values). In order to investigate the influence of the graph, a simple MLP algorithm is also demonstrated in our experiment. When operating our method, we equip our framework with different GCN models (GAT, GCN, and Chebyshev). Comparison with the existing baseline methods is summarized in Table 3.

**Table 3:** Comparison with the existing methods.

Method	Node Classification	Need Features
LP	×	✓
MLP	✓	✓
DeepWalk	×	×
GAT	✓	✓
GCN	✓	✓
Chebyshev	✓	✓
Our	✓	×

### 5.2 Experimental results

We evaluate our method on both datasets: one without node features and the other with node features (results are demonstrated in Tables 4–7). From the result, we can draw some points:

(1) When compared to graph-based methods like LP and DeepWalk, our GCN-based method demonstrates a significant improvement in classification accuracy.
(2) GCN with different inputs demonstrates that features play a crucial role in the GCN node classification problem. Stochastic inputs consistently result in a stochastic output.
(3) When comparing our method with the conventional GCN using different inputs, we conclude that our framework significantly improves classification accuracy.
(4) When conducting the experiments with the full features, it is not surprising to see that conventional GCN models perform better than the proposed approach. The reason is that our method equips the GCN model with a Bayesian framework and a hidden layer constraint term, which is difficult to be optimized.
(5) Comparing the results on the dataset with features to those without features, we find that BGCN with the features can significantly improve classification accuracy.

**Table 4:** Classification accuracy on real graphs without features.

Methods	Citeseer	Cora	Pubmed
LP [65]	0.453	0.680	0.630
MLP+Constant	0.059	0.042	0.103
MLP+Gaussian	0.203	0.308	0.274
DeepWalk [66]	0.432	0.672	0.653
GAT [67]+Constant	0.212	0.316	0.113
GCN [21]+Constant	0.169	0.130	0.181
Chebyshev [20]+Constant	0.228	0.193	0.294
GAT [67]+Gaussian	0.232	0.162	0.262
GCN [21]+Gaussian	0.263	0.285	0.196
Chebyshev [20]+Gaussian	0.219	0.266	0.346
BGCN	**0.542**	**0.705**	**0.726**
BGCN+GAT	0.494	0.576	0.695
BGCN+Chebyshev	0.469	0.678	0.681

**Table 5:** Classification accuracy on real graphs with features.

Methods	Citeseer	Cora	Pubmed
LP [65]	0.453	0.680	0.630
MLP	0.151	0.128	0.326
DeepWalk [66]	0.432	0.672	0.653
GAT [67]	**0.710**	**0.832**	**0.780**
GCN [21]	0.689	0.829	0.779
Chebyshev [20]	0.688	0.786	0.739
	Pseudo Features+Original Features		
BGCN	0.673	0.782	0.642
BGCN+GAT	0.623	0.612	0.567
BGCN+Chebyshev	0.684	0.774	0.662

**Table 6:** Classification accuracy on the image dataset without features. In these datasets, we construct the graph using the k-nearest graph algorithm.

Methods	Extended YaleB	Orl	Yale	Usps	Coil20	Coil100
LP [65]	0.522	0.112	0.581	0.467	0.474	0.612
MLP+Constant	N/A	N/A	N/A	N/A	N/A	N/A
MLP+Gaussian	0.247	0.119	0.483	0.313	0.511	0.045
DeepWalk [66]	0.513	0.101	0.662	0.621	0.703	0.624
GAT [67]+Constant	0.186	0.011	0.354	0.696	0.713	0.103
GCN [21]+Constant	0.206	0.026	0.101	0.136	0.150	0.01
Chebyshev [20]+Constant	0.246	0.079	0.531	0.516	0.683	0.156
GAT [67]+Gaussian	0.332	0.032	0.543	0.575	0.673	0.121
GCN [21]+Gaussian	0.316	0.091	0.616	0.323	0.451	0.117
Chebyshev [20]+Gaussian	0.564	0.131	0.554	0.664	0.763	0.594
BGCN	0.492	**0.133**	**0.738**	**0.859**	0.841	0.771
BGCN+GAT	0.574	0.102	0.701	0.851	0.772	0.823
BGCN+Chebyshev	**0.616**	0.131	0.646	**0.859**	**0.882**	**0.842**

**Table 7:** Classification accuracy on the image dataset with features. In these datasets, we construct the graph using the k-nearest graph algorithm.

Methods	Extended YaleB	Orl	Yale	Usps	Coil20	Coil100
LP [65]	0.522	0.112	0.581	0.467	0.474	0.612
MLP	**0.798**	0.150	**0.799**	0.709	0.829	0.669
DeepWalk [66]	0.424	0.035	0.364	0.621	0.703	0.422
GAT [67]	0.656	0.132	0.682	0.891	0.954	0.704
GCN [21]	0.646	0.067	0.646	**0.940**	0.901	0.649
Chebyshev [20]	0.713	**0.154**	0.712	0.852	**1.000**	**0.721**
	Pseudo Features+Original Features					
BGCN	0.733	0.068	0.664	0.922	0.911	0.643
BGCN+GAT	0.631	0.128	0.646	0.842	0.925	0.665
BGCN+Chebyshev	0.697	0.150	0.609	0.934	0.972	0.597

### 5.3 Effect of algorithm parameters

In this subsection, we investigate the influence of algorithm parameters under different algorithm settings.

**Fig 7:**
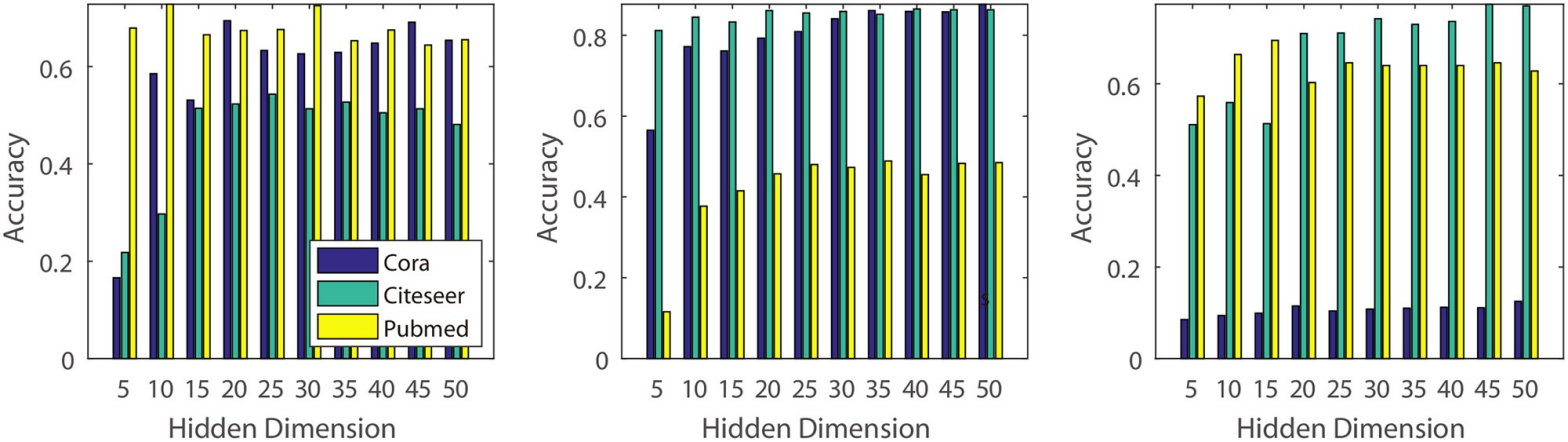
Parameter effect. Illustration of the effect of the pseudo features’ hidden dimension. The x-axis representsthe dimension of the pseudo features’ hidden space, while the Y-axis corresponds to the classification accuracy.

**Fig 8:**
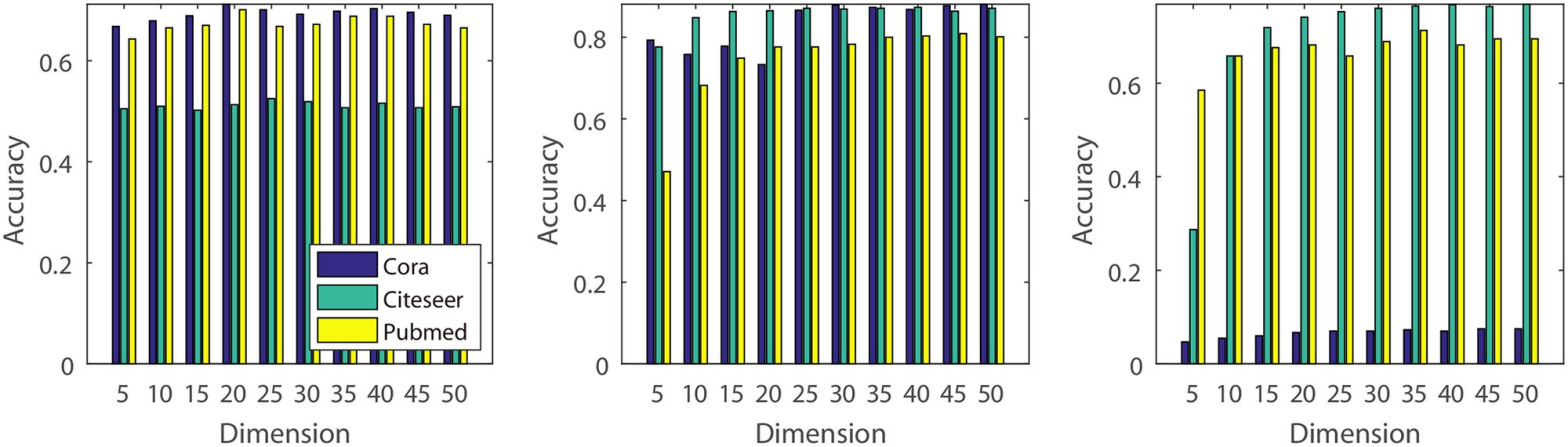
Parameter effect. Illustration of the effect of the GCN hidden unit number. The x-axis represents the GCN hidden unit dimension, and the y-axis represents the classification accuracy.

**Fig 9:**
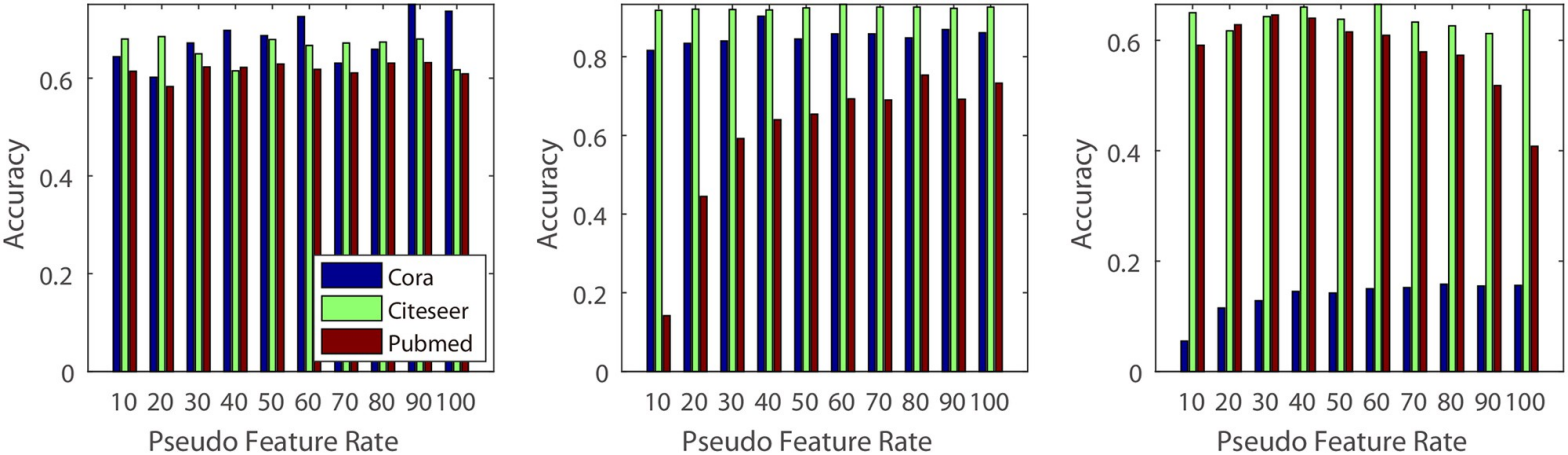
Parameter effect. Illustration of the effect of the rate of pseudo features: X-axis represents the rate of pseudo features’ dimension, and Y-axis represents the classification accuracy.

**Fig 10:**
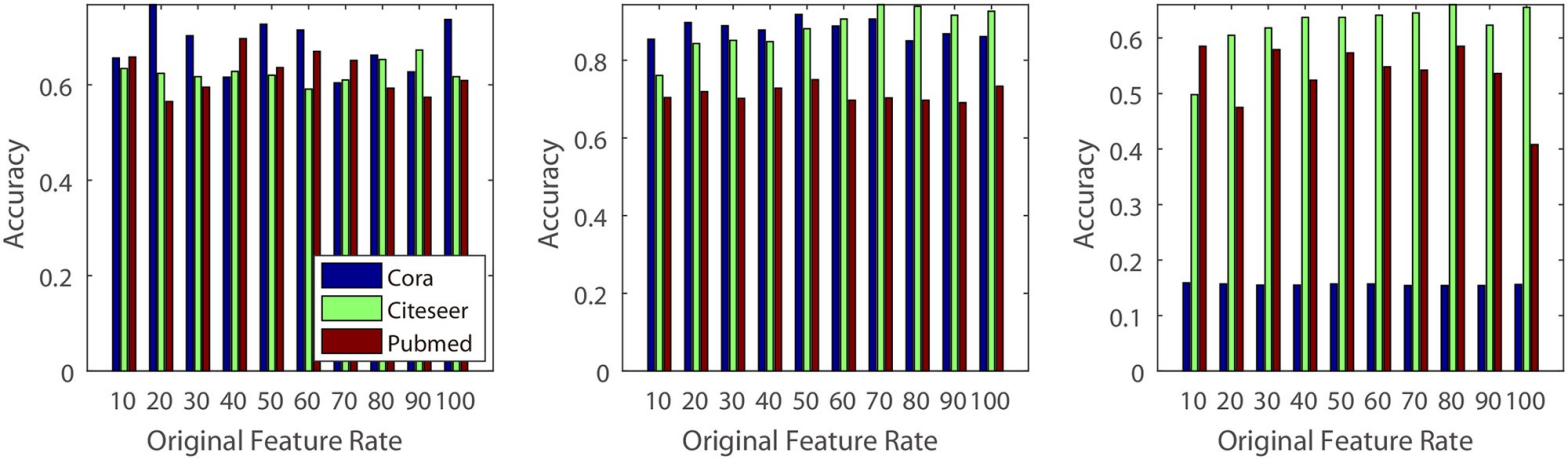
Parameter effect. Illustration of the effect of original features. The X-axis represents the rate of the original features’ dimension, and the Y-axis represents the classification accuracy.

**(1) Dimension of the pseudo features’ hidden variable**: In this experiment, we vary the dimension of the hidden variable from 10 to 40 and conduct experiments on five different real datasets (Fig 7). The experimental results indicate that small values may lead to decreased classification accuracy. This could be because smaller dimensions contain less information compared to larger values, which can capture more detailed information about the original graph structure.
**(2) Number of the GCN hidden units**: Similar to the experiments on dimension effects, we investigated the impact of GCN hidden units using different values. Specifically, we varied the value from 10 to 40 (Fig 8). From the result, we know that, different to the Dimension of the hidden variable, classification accuracy of the BGCN is not sensitive to the hidden units number in the citeseer and cora, and increase the classification accuracy in the pubmed, usps and coil20 dataset. The reason may be that pubmed, usps and coil20 datasets are much more complicated than the citeseer and cora, and require a much more complicated model.
**(3) Rate of Original and Pseudo Features**: For the algorithm with original features, we construct additional experiments with various rate of original and pseudo feature dimensions. In our experiments, we decrease the rate of original and pseudo features dimension from 100% to 10% (Figs 9 and 10). From the experimental results, we know that some datasets decrease their classification accuracy when the rate of original features is decreasing. The reason is that original features contain much more information that may not be simulated by the pseudo features. Additionally, for the pseudo features, we also observe that classification accuracy decreases when the rate of pseudo features decreases. The reason is that pseudo features contain much more information than the original features for some datasets.

### 5.4 Convergence analysis

**Fig 11:**
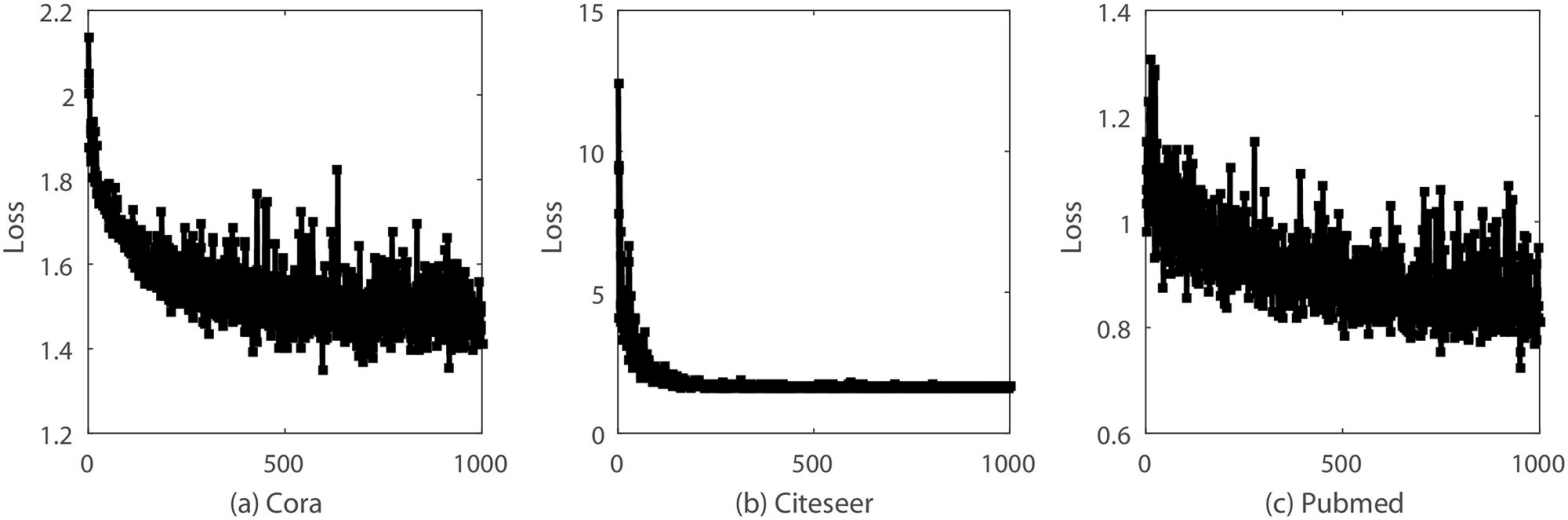
Convergence analysis. Loss curve of the BGCN. (a) is the cora dataset. (b) is the citerseer dataset. (c) is the pubmed dataset.

We evaluate the convergence of Algorithm 1 on real graph datasets (Cora, Citeseer, Pubmed). We show the convergence curve in Fig 11. From the figure, we can draw a conclusion that our model converges after 500 iterations. We also find that the loss curve is stable in the citeseer dataset and unstable in the cora and pubmed dataset. The reason is that cora and pubmed are much more complicated dataset than the cora dataset (as evident from the classification accuracy in Tables 6 and 7, where the accuracy for Cora and Pubmed is lower than that for Citeseer.).

## 6 Conclusions

In this paper, we extend the application scope of the Graph Convolutional Network. Different from the conventional GCN methods which require the features in the input space, our method equips the GCN input with the generated pseudo features, and assumes that the labels are generated from the GCN with a Bayesian framework and graph constraint. Experiments with the graph constraint generation features demonstrate some facts that: (1) random generation feature could benefit the graph node classification without features; (2) graph constraint feature generation method is another boost for the classification accuracy. There are also some spaces for the further study: (1) extending the proposed graph constraint feature generation model to the unsupervised learning framework; (2) our model is designed for the static graph. For the future work, we could extend it to the dynamic situation; (3) although our model is able to handle the different graph applications, it requires the eigenvalue decomposition which is time cost. Thus, a fast eigenvalue decomposition will be a plus. Additionally, for the real system, our model can be used to replace the conventional GCN model as plug and play modules.

## References

[1] Hu X, Liu Z, Zhou H, Fang J, Lu H. Deep HT: A deep neural network for diagnose on MR images of tumors of the hand. PLOS ONE. 2020;15(8):1–13. doi: 10.1371/journal.pone.0237606 PMC7428075 32797089

[2] Ruiz Puentes P, Valderrama N, González C, Daza L, Muñoz-Camargo C, Cruz JC, et al. PharmaNet: Pharmaceutical discovery with deep recurrent neural networks. PLOS ONE. 2021;16(4):1–22. doi: 10.1371/journal.pone.0241728 33901196 PMC8075191

[3] Law MT, Urtasun R, Zemel RS. Deep spectral clustering learning. In: International Conference on Machine Learning; 2017. p. 1985–1994.

[4] Gatys LA, Ecker AS, Bethge M. Image Style Transfer Using Convolutional Neural Networks. In: Computer Vision & Pattern Recognition; 2016.

[5] Nguyen T, Le H, Quinn TP, Nguyen T, Le TD, Venkatesh S. GraphDTA: predicting drug–target binding affinity with graph neural networks. Bioinformatics. 2021;37(8):1140–1147. doi: 10.1093/bioinformatics/btaa921 33119053

[6] You J, Liu B, Ying Z, Pande V, Leskovec J. Graph convolutional policy network for goal-directed molecular graph generation. Advances in neural information processing systems. 2018;31.

[7] Johnson R, Li MM, Noori A, Queen O, Zitnik M. Graph Artificial Intelligence in Medicine. Annual Review of Biomedical Data Science. 2024;7. 38749465 10.1146/annurev-biodatasci-110723-024625 PMC11344018

[8] Sun H, Liu Z, Wang S, Wang H. Adaptive Attention-Based Graph Representation Learning to Detect Phishing Accounts on the Ethereum Blockchain. IEEE Transactions on Network Science and Engineering. 2024;. doi: 10.1109/TNSE.2024.3355089

[9] Liu Z, Yang D, Wang Y, Lu M, Li R. EGNN: Graph structure learning based on evolutionary computation helps more in graph neural networks. Applied Soft Computing. 2023;.

[10] Zhou Y, Huo H, Hou Z, Bu F. A deep graph convolutional neural network architecture for graph classification. PLOS ONE. 2023;18(3):1–31. doi: 10.1371/journal.pone.0279604 36897837 PMC10004633

[11] Jeong H, Cho YR, Gim J, Cha SK, Kim M, Kang DR. GraphMHC: Neoantigen prediction model applying the graph neural network to molecular structure. PLOS ONE. 2024;19(3):1–18. doi: 10.1371/journal.pone.0291223 38536842 PMC10971776

[12] Zhou J, Cui G, Zhang Z, Yang C, Liu Z, Wang L, et al. Graph Neural Networks: A Review of Methods and Applications. arXiv: Learning. 2018;.

[13] Li ZL, Zhang GW, Yu J, Xu LY. Dynamic graph structure learning for multivariate time series forecasting. Pattern Recognition. 2023;138:109423. doi: 10.1016/j.patcog.2023.109423

[14] Franceschi L, Niepert M, Pontil M, He X. Learning discrete structures for graph neural networks. In: International conference on machine learning. PMLR; 2019. p. 1972–1982.

[15] Atwood J, Towsley D. Diffusion-Convolutional Neural Networks. NIPS. 2015;.

[16] Duvenaud D, Maclaurin D, Aguileraiparraguirre J, Gómezbombarelli R, Hirzel T, Aspuruguzik A, et al. Convolutional Networks on Graphs for Learning Molecular Fingerprints. In: NIPS; 2015.

[17] Niepert M, Ahmed M, Kutzkov K. Learning Convolutional Neural Networks for Graphs. ICML. 2016;.

[18] Monti F, Boscaini D, Masci J, Rodola E, Svoboda J, Bronstein MM. Geometric Deep Learning on Graphs and Manifolds Using Mixture Model CNNs. CVPR. 2017; p. 5425–5434.

[19] Bruna J, Zaremba W, Szlam A, Lecun Y. Spectral Networks and Locally Connected Networks on Graphs. ICLR. 2014;.

[20] Defferrard M, Bresson X, Vandergheynst P. Convolutional neural networks on graphs with fast localized spectral filtering. NIPS. 2016; p. 3844–3852.

[21] Kipf TN, Welling M. Semi-supervised classification with graph convolutional networks. ICLR. 2017;.

[22] Jiang B, Zhang Z, Lin D, Tang J, Luo B. Semi-Supervised Learning With Graph Learning-Convolutional Networks. In: CVPR; 2019. p. 11313–11320.

[23] Chen Y, Wu L, Zaki M. Iterative deep graph learning for graph neural networks: Better and robust node embeddings. Advances in neural information processing systems. 2020;33:19314–19326.

[24] Tang J, Hu W, Gao X, Guo Z. Joint learning of graph representation and node features in graph convolutional neural networks. arXiv preprint arXiv:190904931. 2019;.

[25] Gan J, Hu R, Mo Y, Kang Z, Peng L, Zhu Y, et al. Multigraph Fusion for Dynamic Graph Convolutional Network. IEEE Transactions on Neural Networks and Learning Systems. 2024;35(1):196–207. doi: 10.1109/TNNLS.2022.3172588 35576414

[26] Zhao J, Wang X, Shi C, Hu B, Song G, Ye Y. Heterogeneous Graph Structure Learning for Graph Neural Networks. Proceedings of the AAAI Conference on Artificial Intelligence. 2019;35(5).

[27] Yujun C, Liuhao G, Jun L, Jianfei C, Tat-Jen C, Junsong Y, et al. Exploiting Spatial-temporal Relationships for 3D Pose Estimation via Graph Convolutional Networks. In: ICCV; 2019.

[28] Yan S, Xiong Y, Lin D. Spatial temporal graph convolutional networks for skeleton-based action recognition. In: AAAI; 2018.

[29] Huang L, Huang Y, Ouyang W, Wang L. Part-Level Graph Convolutional Network for Skeleton-Based Action Recognition. In: Computer Vision & Pattern Recognition; 2020.

[30] Feng L, Zhao Y, Zhao W, Tang J. A comparative review of graph convolutional networks for human skeleton-based action recognition. Artificial Intelligence Review. 2022; p. 1–31.

[31] Yang L, Zhan X, Chen D, Yan J, Loy CC, Lin D. Learning to Cluster Faces on an Affinity Graph. CVPR. 2019;.

[32] Wang Z, Zheng L, Li Y, Wang S. Linkage Based Face Clustering via Graph Convolution Network. CVPR. 2019;.

[33] Tsitsulin A, Palowitch J, Perozzi B, Müller E. Graph clustering with graph neural networks. Journal of Machine Learning Research. 2023;24(127):1–21.

[34] Liu Y, Yang X, Zhou S, Liu X, Wang S, Liang K, et al. Simple contrastive graph clustering. IEEE Transactions on Neural Networks and Learning Systems. 2023;. 37368805 10.1109/TNNLS.2023.3271871

[35] Zhang Z, Zhang Y, Feng R, Zhang T, Fan W. Zero-Shot Sketch-Based Image Retrieval via Graph Convolution Network. Proceedings of the AAAI Conference on Artificial Intelligence. 2020;34(7):12943–12950.

[36] Chen J, Pan L, Wei Z, Wang X, Chua TS. Zero-Shot Ingredient Recognition by Multi-Relational Graph Convolutional Network. Proceedings of the AAAI Conference on Artificial Intelligence. 2020;34(7):10542–10550.

[37] Ru X, Moore JM, Zhang XY, Zeng Y, Yan G. Inferring patient zero on temporal networks via graph neural networks. In: Proceedings of the AAAI Conference on Artificial Intelligence. vol. 37; 2023. p. 9632–9640.

[38] Zhu X, Yang J, Zhang C, Zhang S. Efficient utilization of missing data in cost-sensitive learning. IEEE Transactions on Knowledge and Data Engineering. 2019; p. 1–1. doi: 10.1109/TKDE.2019.2951103

[39] Van Buuren S. Flexible imputation of missing data. CRC press; 2018.

[40] Azur MJ, Stuart EA, Frangakis C, Leaf PJ. Multiple imputation by chained equations: what is it and how does it work? International journal of methods in psychiatric research. 2011;20(1):40–49. doi: 10.1002/mpr.329 21499542 PMC3074241

[41] Yang Q, Ling C, Chai X, Pan R. Test-Cost Sensitive Classification on Data with Missing Values. IEEE Transactions on Knowledge & Data Engineering. 2006;18(5):626–638. doi: 10.1109/TKDE.2006.84

[42] Spinelli I, Scardapane S, Uncini A. Missing data imputation with adversarially-trained graph convolutional networks. Neural Networks. 2020;. doi: 10.1016/j.neunet.2020.06.005 32563022

[43] Acuna E, Rodriguez C. The treatment of missing values and its effect on classifier accuracy. In: Classification, clustering, and data mining applications. Springer; 2004. p. 639–647.

[44] Dick U, Haider P, Scheffer T. Learning from incomplete data with infinite imputations. In: Proceedings of the 25th international conference on Machine learning; 2008. p. 232–239.

[45] Lakshminarayan K, Harp SA, Goldman RP, Samad T, et al. Imputation of Missing Data Using Machine Learning Techniques. In: KDD; 1996. p. 140–145.

[46] Zhang W. Association-based multiple imputation in multivariate datasets: A summary. In: Proceedings of 16th International Conference on Data Engineering. IEEE Computer Society; 2000. p. 310–310.

[47] Peng CYJ, Zhu J. Comparison of two approaches for handling missing covariates in logistic regression. Educational and Psychological Measurement. 2008;68(1):58–77. doi: 10.1177/0013164407305582

[48] Yoon J, Jordon J, Van Der Schaar M. Gain: Missing data imputation using generative adversarial nets. ICML. 2018;.

[49] Nazabal A, Olmos PM, Ghahramani Z, Valera I. Handling incomplete heterogeneous data using vaes. Pattern Recognition. 2020; p. 107501. doi: 10.1016/j.patcog.2020.107501

[50] Wen J, Liu C, Deng S, Liu Y, Fei L, Yan K, et al. Deep double incomplete multi-view multi-label learning with incomplete labels and missing views. IEEE Transactions on Neural Networks and Learning Systems. 2023;. 37030862 10.1109/TNNLS.2023.3260349

[51] Sun Y, Li J, Xu Y, Zhang T, Wang X. Deep learning versus conventional methods for missing data imputation: A review and comparative study. Expert Syst Appl. 2023;227:120201. doi: 10.1016/j.eswa.2023.120201

[52] Kingma DP, Welling M. Stochastic gradient VB and the variational auto-encoder. In: Second International Conference on Learning Representations, ICLR. vol. 19; 2014.

[53] Mao Y, Zhang J, Xiang M, Zhong Y, Dai Y. Multimodal variational auto-encoder based audio-visual segmentation. In: Proceedings of the IEEE/CVF International Conference on Computer Vision; 2023. p. 954–965.

[54] Shin Y, Yoo KM, Lee SG. Utterance Generation With Variational Auto-Encoder for Slot Filling in Spoken Language Understanding. IEEE Signal Processing Letters. 2019;PP(99):1–1.

[55] Tang D, Liang D, Jebara T, Ruozzi N. Correlated Variational Auto-Encoders. ICML. 2019;.

[56] Mathieu E, Lan CL, Maddison CJ, Tomioka R, Teh YW. Continuous Hierarchical Representations with Poincare Variational Auto-Encoders. NeurIPS. 2019; p. 12544–12555.

[57] Hinton GE, Osindero S, Teh YW. A fast learning algorithm for deep belief nets. Neural computation. 2006;18(7):1527–1554. doi: 10.1162/neco.2006.18.7.1527 16764513

[58] Hinton GE, Salakhutdinov RR. Reducing the dimensionality of data with neural networks. science. 2006;313(5786):504–507. doi: 10.1126/science.1127647 16873662

[59] Nair V, Hinton GE. Rectified linear units improve restricted boltzmann machines. In: ICML; 2010.

[60] Goodfellow I, Pouget-Abadie J, Mirza M, Xu B, Warde-Farley D, Ozair S, et al. Generative adversarial nets. In: Advances in neural information processing systems; 2014. p. 2672–2680.

[61] Arjovsky M, Chintala S, Bottou L. Wasserstein Generative Adversarial Networks. vol. 70 of Proceedings of Machine Learning Research. International Convention Centre, Sydney, Australia: PMLR; 2017. p. 214–223.

[62] Bengio Y, Laufer E, Alain G, Yosinski J. Deep generative stochastic networks trainable by backprop. In: International Conference on Machine Learning; 2014. p. 226–234.

[63] Gholami B, Pavlovic V. Probabilistic Temporal Subspace Clustering. In: Proceedings of the IEEE Conference on Computer Vision and Pattern Recognition; 2017. p. 3066–3075.

[64] Sen P, Namata G, Bilgic M, Getoor L, Gallagher B, Eliassirad T. Collective Classification in Network Data. Ai Magazine. 2008;29(3):93–106. doi: 10.1609/aimag.v29i3.2157

[65] Zhu X, Lafferty J, Ghahramani Z. Combining Active Learning and Semi-Supervised Learning Using Gaussian Fields and Harmonic Functions. ICML. 2003;.

[66] Perozzi B, Al-Rfou R, Skiena S. DeepWalk: Online Learning of Social Representations. In: Acm Sigkdd International Conference on Knowledge Discovery & Data Mining; 2014.

[67] Veličković P, Cucurull G, Casanova A, Romero A, Lio P, Bengio Y. Graph attention networks. ICLR. 2018;.

